# A transcriptomic reevaluation of the accessory olfactory organ in Bichir (*Polypterus senegalus*)

**DOI:** 10.1186/s40851-022-00189-z

**Published:** 2022-02-08

**Authors:** Atsuhiro Sakuma, Zicong Zhang, Eri Suzuki, Tatsuki Nagasawa, Masato Nikaido

**Affiliations:** 1 School of Life Science and Technology, Tokyo Institute of Technology, Meguro-ku, Tokyo, 152-8550 Japan; 2 Institute for the Advanced Study of Human Biology, Kyoto University, Sakyo-ku, Kyoto, 606-8501 Japan

## Abstract

Fish possess one olfactory organ called the olfactory epithelium (OE), by which various chemical substances are detected. On the other hand, tetrapods possess two independent olfactory organs called the main olfactory epithelium (MOE) and vomeronasal organ (VNO), each of which mainly detects general odorants and pheromones, respectively. Traditionally, the VNO, so-called concentrations of vomeronasal neurons, was believed to have originated in tetrapods. However, recent studies have identified a primordial VNO in lungfish, implying that the origin of the VNO was earlier than traditionally expected. In this study, we examined the presence/absence of the VNO in the olfactory organ of bichir (*Polypterus senegalus*), which is the most ancestral group of extant bony vertebrates. In particular, we conducted a transcriptomic evaluation of the accessory olfactory organ (AOO), which is anatomically separated from the main olfactory organ (MOO) in bichir. As a result, several landmark genes specific to the VNO and MOE in tetrapods were both expressed in the MOO and AOO, suggesting that these organs were not functionally distinct in terms of pheromone and odorant detection. Instead, differentially expressed gene (DEG) analysis showed that DEGs in AOO were enriched in genes for cilia movement, implying its additional and specific function in efficient water uptake into the nasal cavity other than chemosensing. This transcriptomic study provides novel insight into the long-standing question of AOO function in bichir and suggests that VNO originated in the lineage of lobe-finned fish during vertebrate evolution.

**Supplementary Information:**

The online version contains supplementary material available at 10.1186/s40851-022-00189-z.

## Background

Pheromones are chemical substances released by an individual and received by another individual of the same species, eliciting innate social and sexual behaviors. In tetrapods, such as amphibians, reptiles and mammals, pheromones are predominantly detected by the vomeronasal organ (VNO), in which vomeronasal sensory neurons are concentrated. The VNO is anatomically distinct from the main olfactory epithelium (MOE), by which general odorants are detected. Neurons of the MOE project their axons to the main olfactory bulb (MOB), whereas those in the VNO project their axons to the accessory olfactory bulb (AOB) [1, 2]. Namely, the olfactory organs of tetrapods are divided into two functionally distinct organs, MOE and VNO. In contrast, the olfactory organ of teleost fishes is not differentiated into the MOE and VNO but consists solely of the OE [3]. Since the VNO exists in tetrapods but not in teleost fish, the VNO was believed to have originated with adaptation to the terrestrial environment [4, 5]. Recently, however, the recess epithelium (RecE), which is expected to be a primordial VNO, was found in the olfactory organ of lungfish, a lobe-finned fish. This finding implies that the VNO originated prior to terrestrial adaptation in vertebrate evolution [6–8].

In mammals, two types of seven transmembrane G-protein-coupled receptor genes were shown to be expressed by microvillous neurons of the VNO, namely, vomeronasal receptor type I (*V1R*) [9] and vomeronasal receptor type II (*V2R*) [10], both of which form multigene families. *V1R*s and *V2R*s are predominantly expressed in the VNO, whereas another seven transmembrane G-protein-coupled receptor genes are predominantly expressed by ciliated neurons of the MOE, namely, olfactory *receptors (ORs*) [11] and trace amine-associated receptors (*TAARs*) [12]. In teleost fish, the expression patterns of these four receptor families are similar to those of mammals in that *V1R*s and *V2R*s are expressed by microvillous neurons and *OR*s and *TAAR*s by ciliated neurons. However, they are all collectively expressed in the OE [13–16]. In microvillous neurons, *V1R*s are coexpressed with *Gi*_*2*_, a specific type of G-protein gene, and *V2R*s are coexpressed with *Go* [9, 17, 18], both of which are further coexpressed with the transient receptor potential cation channel, subfamily C, member 2 (*TRPC2*) gene [19, 20]. In ciliated neurons, *OR*s and *TAAR*s are coexpressed with the *Golf* and cyclic nucleotide gated channel subunit alpha 2 (*CNGA2*) genes [21]. The neuron types and gene coexpression patterns are shared among tetrapods and teleost fish regardless of the presence/absence of VNO [22, 23]. Previous bioinformatic analyses revealed that the genetic components of vomeronasal sensory neurons are conserved in a broad range of vertebrates from lampreys to mammals [24]. In this study, we traced the evolutionary history of the VNO in vertebrates based on the expression patterns of several landmark genes of vomeronasal and olfactory sensory neurons.

Recent comprehensive phylogenetic analyses on vomeronasal genes have also revealed that the *V1R* and *V2R* families are further subdivided into two major groups, the “tetrapod-type” (t-*V1R*, t-*V2R*) and “fish-type” (f-*V1R*, f-*V2R*) ([25], Zhang et al. 2021 in press). The “tetrapod-type” denotes that they were originally identified in mammals and that the “fish-type” was originally identified in teleost fish, each of which is distinct in the phylogenetic tree. t-*V1R*s and t-*V2R*s and f-*V1R*s and f-*V2R*s are specific to tetrapods and teleost fish, respectively, with some exceptions: t-*V1R*s and t-*V2R*s were also found in coelacanths [26, 27] and several basal ray-finned fishes ([28], Zhang et al. 2021 in press), implying that their origin predated terrestrial adaptation. In addition, a novel member of the V1R family, *ancV1R,* was recently identified [29]. Importantly, *ancV1R* is expressed by all vomeronasal sensory neurons of the VNO. The expression pattern of *ancV1R* is distinct from that of conventional *V1R*s, which show sparse expression patterns following one neuron-one receptor rule [30, 31]. Similar to *ancV1R*, *V2R2* was also shown to be expressed by all vomeronasal sensory neurons [32, 33].

**Fig. 1:**
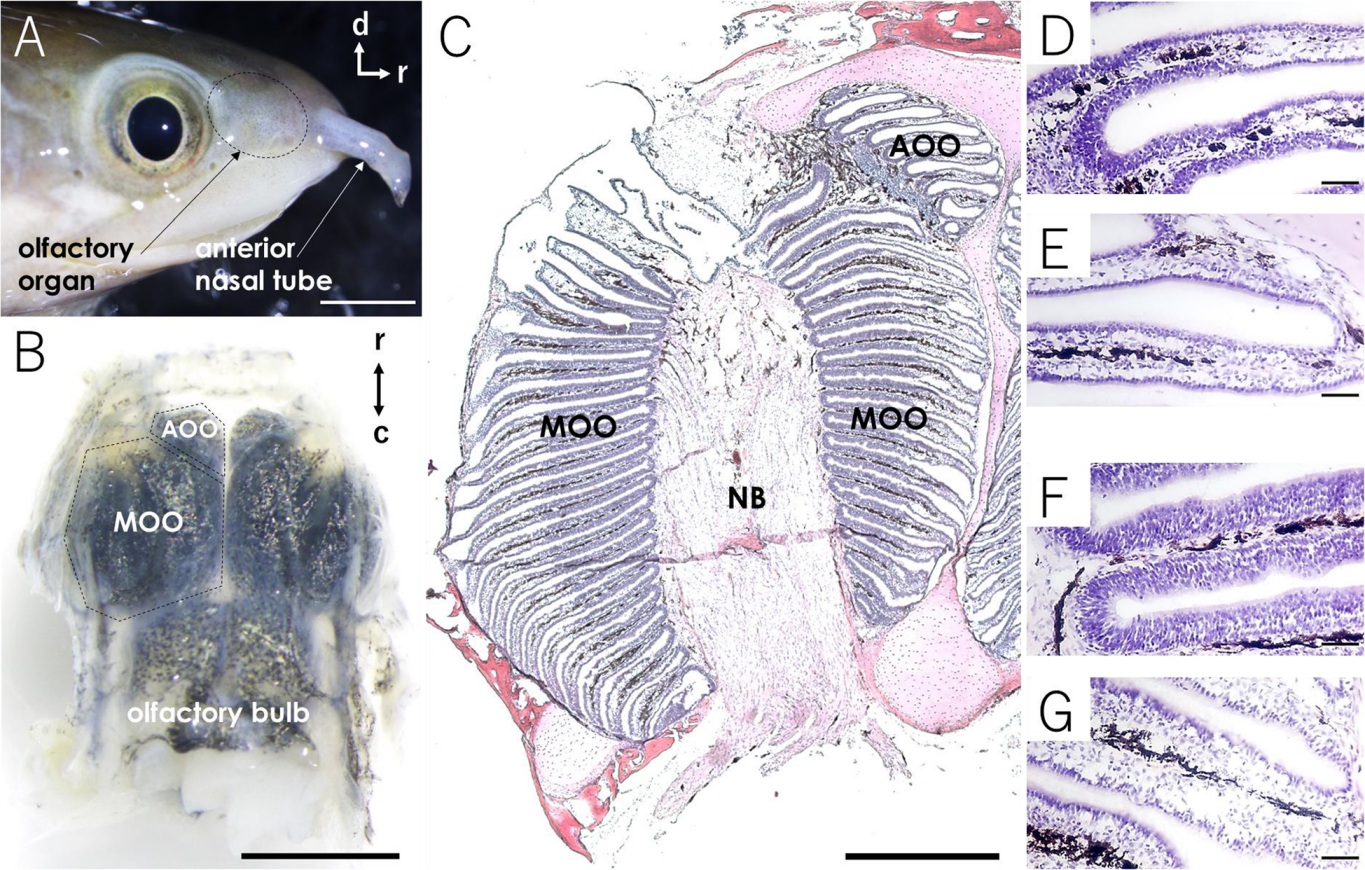
Structure of the olfactory organ of bichir (*Polypterus senegalus*). (**A**) Lateral view of the head tip of bichir. The olfactory organ is located in the dotted circle between the eye and the anterior nasal tube. (**B**) The olfactory organ and part of the brain removed from the head. The left and right olfactory organs are composed of two structures: the MOO and AOO, respectively. The caudal posterior end of the MOO is connected to the olfactory bulb. (**C**) Overall view of a HE-stained horizontal section of the olfactory organ. The lamellae of olfactory organs were stained blue–violet, and the nerve bundles (NB) and cartilage were stained red–violet. Higher magnification views of the thickened (**D**, **F**) and thinner (**E**, **G**) epithelium lining the AOO and MOO lamellae, showing sensory and nonsensory epithelium, respectively. Scale bars indicate 3 mm (**A**, **B**), 1mm (**C**), or 50 μm (**D**-**G**). d: dorsal, r: rostral, c: caudal

The olfactory organ of the most basal group of extant ray-finned fish “bichir” possesses a unique structure, which is subdivided into two organs, the main olfactory organ (MOO) and accessory olfactory organ (AOO), although the functional differences between these two organs remain unknown (Fig. 1, [34]). The existence of anatomically distinct organs in bichir reminds us of the idea that these two organs may correspond to the MOE and VNO in tetrapods. Examining whether a primordial VNO originated even earlier than lungfish is of primary importance in the field of chemosensory evolution of vertebrates. Thus, we conducted a transcriptional re-evaluation of AOO and MOO in bichir by examining the expression of the genetic components of VNO and MOE, such as *V1R*s, *V2R*s, *Go*, *Gi*_*2*_, *TRPC2*, *ancV1R*, *V2R2*, *Golf*, and *CNGA2*, and by performing differentially expressed gene (DEG) analyses. As a result, vomeronasal and olfactory sensory neurons were distributed in both the AOO and MOO, suggesting that they are functionally undifferentiated in terms of pheromone and odorant detection. Instead, the results of the DEG analyses implied that AOO has an additional function other than chemosensing, namely, its ability to take up water into the nasal cavity efficiently. Our transcriptomic study suggests that the common ancestor of bony vertebrates already possessed vomeronasal sensory neurons typical of tetrapods, but the VNO originated later in the lineage of lobe-finned fish.

## Materials and methods

### Animals and histological observation

For all animal samples, body length was measured from snout to tail fin. The 11–25 cm bichirs (*Polypterus senegalus*) and the 30 cm lungfish (*Protopterus annectens*) used for the preparation of frozen sections of olfactory organs and total RNA extractions were purchased from a commercial supplier and kept under standard conditions suitable for tropical fish breeding until the experimental manipulations. The 15 cm spotted gar (*Lepisosteus oculatus*) used for DNA extraction was purchased from a commercial supplier and kept under the above conditions until the experimental manipulations. The bichirs and lungfish were anesthetized on ice before euthanizing by decapitation. The spotted gar was cut off a part of its fin and returned to the breeding conditions described above. All experimental manipulations using the animals were conducted at the Tokyo Institute of Technology with the approval of the university committee. PFA-fixed head samples of 27.5–34 cm spotted gars used for the preparation of frozen sections of the olfactory organ were provided by the Laboratory of Physiology, Atmosphere and Ocean Research Institute, University of Tokyo. The olfactory organs removed from the head of bichirs or lungfish were fixed in 4% PFA/0.7x PBS overnight at 4 °C. The PFA-fixed olfactory organs were replaced with sucrose overnight at 4 °C in 20% sucrose/0.7x PBS solution. The sucrose-replaced olfactory organs were embedded in O.C.T. compund (Sakura Finetek) and frozen in liquid nitrogen. From the frozen blocks of the olfactory organs, 14 μm thick frozen sections were prepared using a cryostat. These frozen sections were stored at − 80 °C until use.

Frozen sections of the bichir olfactory organs were brought to room temperature and washed with tap water and distilled water to remove the O.C.T. compound. Each section was stained with hematoxylin for 4 min and washed with tap water. The sections were then stained with eosin for 10 min and treated with 70% ethanol for 1 min, 80% ethanol for 1 min, 90% ethanol for 1 min, and 100% ethanol for 5 min three times. The sections were then treated with xylene for 5 min three times and sealed in ENTELLAN NEW (MERCK).

### Transcriptome analyses

Total RNA used for transcriptome analysis was extracted from the olfactory organs of three individual bichirs and one individual lungfish using TRIzol (Invitrogen) or TRI Reagent (Molecular Research Center). The olfactory organs of bichirs were separated into MOO and AOO. Extracted RNA samples were stored at − 30 °C until use. All RNA sequencing was performed using Illumina NovaSeq6000 sequencers after constructing sequence libraries using the TruSeq Stranded mRNA Library Kit (Illumina). The length of each read was 101 bp, and the layout was paired end. The total number of reads obtained is as follows. MOO_1: 43,966,110; MOO_2: 51,993,914; MOO_3: 41,642,906; AOO_1: 41,003,732; AOO_2: 59,561,386; AOO_3: 52,225,820; lungfish olfactory organ: 55,285,188. All sequence reads were deposited in the DDBJ Sequence Read Archive under accession nos. PRJDB12173 and PRJDB12387. The RNA-seq reads of lungfish were assembled de novo using Trinity (ver. 2.4.0) [35, 36] or Bridger (ver. 2014-12-01) [37]. The RNA-seq reads of bichirs were mapped to their genome sequence (https://ftp.ncbi.nlm.nih.gov/genomes/all/GCF/016/835/505/GCF_016835505.1_ASM1683550v1/GCF_016835505.1_ASM1683550v1_genomic.fna.gz) using Bowtie2 (ver. 2.3.5.1) [38] for the calculation of the expected counts of RNA-seq reads mapped to the genome sequence. The expected counts were normalized to RPM and used for differential expression gene (DEG) analyses via multiple comparison test against the results of each three samples of the MOOs and AOOs using TCC (ver. 1.24.0) [39]. The detected DEGs were annotated by genome annotation data (https://ftp.ncbi.nlm.nih.gov/genomes/all/GCF/016/835/505/GCF_016835505.1_ASM1683550v1/GCF_016835505.1_ASM1683550v1_genomic.gff.gz). The annotated DEGs were subjected to GO analysis using WebGestalt (Web-based GEne SeT AnaLysis Toolkit: http://www.webgestalt.org).

In addition, several landmark genes of the VNO and MOE in tetrapods (*ancV1R*, *TRPC2*, *CNGA2*, *Gi2*, *Go*, and *Golf*) were manually identified and annotated by tblastn searches against the bichir genome using the corresponding sequences of mice (*Mus musculus*) in Ensembl (https://asia.ensembl.org/Mus_musculus/Info/Index) as queries. The sequences of *V1R*s and *V2R*s were obtained by homology search against the genome sequences of bichir using our original software, FATE (ver.2.7.1, https://github.com/Hikoyu/FATE). *V1R*s and *V2R*s identified in previous studies were used as queries ([26, 28], Zhang et al. 2021 in press). All of the resultant amino acid sequences of *V1R*s and *V2R*s were aligned using MAFFT (ver. 7.475) [40]. The phylogenetic trees were constructed by the maximum likelihood method using RAxML (ver. 8.2.12) [41] under the best fit model estimated by modeltest (http://evomics.org/resources/software/molecular-evolution-software/modeltest/) implemented in MEGAX [42]. The phylogenetic trees were visualized by FigTree (ver. 1.4.4, http://tree.bio.ed.ac.uk/software/figtree/).

### Fluorescence in situ hybridization (FISH) of the olfactory organ of bichir

Using the extracted total RNA from the olfactory organs of bichir and lungfish as a template, cDNA was synthesized by reverse transcription reaction using SuperScript III RTase (Invitrogen). Each of the gene fragments was amplified by PCR using genomic DNA or the synthesized cDNA as a template using the primers shown in Supplementary Table S1. The amplified PCR products were cloned using the pGEM-T-Vector (Promega) and *E. coli* of the DH5α strain. The sequences of the cloned PCR products were confirmed by sequencing using the Sanger method. All genetic recombination experiments using *E. coli* were conducted at the Tokyo Institute of Technology with the approval of the university committee. The plasmid vectors containing the cloned gene sequences were extracted from the *E. coli* colonies and cut using appropriate restriction enzymes. Digoxigenin- or fluorescein-labeled RNA probes were synthesized using the cut plasmid vector as a template in the presence of T7 or SP6 RNA Polymerase (Roche) and DIG or FITC RNA labeling mix (Roche). These probes were stored at − 30 °C until use.

In single color FISH, the frozen sections were brought to room temperature and treated with 4% PFA/0.7x PBS for 5 min, 0.3% H_2_O_2_ / 0.7x PBS for 15 min, 10 mg/ml proteinase K/0.7x PBS for 10 min at 37 °C, 4% PFA/0.7x PBS for 10 min, 0.2% glycine/0.7x PBS for 5 min, and 0.2 N HCl for 20 min. Acetylation reaction was conducted by steering in 0.1 M triethanolamine-HCl solution for 5 min while adding 1 ml of acetic anhydride drop by drop. Prehybridization was conducted by treatment with hybridization solution containing 50% formamide, 0.01M Tris-HCl (pH 7.5), 0.2 mg/ml Yeast tRNA, 5% dextran sulfate, 1x Denhardt's reagent, 0.6M NaCl, 2.5% SDS, 0.001M EDTA (pH 8.0) for 30 min. Hybridization was conducted in 2.5 ng/μl DIG or FITC-labeled RNA probe/hybridization solution overnight at 60 °C. After hybridization, the sections were washed with 5x SSC and treated twice with 5x SSC/50% formamide for 15 min at 50 °C. After treatment with 2 μg/ml RNase A (Sigma)/TNE at 37 °C for 30 min, the sections were washed twice with 2x SSC and 0.2x SSC each for 15 min at 50 °C. Endogenous biotin was blocked with a Streptavidin/Biotin Blocking Kit (VECTOR) and treated with blocking solution containing 1% blocking reagent (Kiko Tech) in TBS for 60 min. The antibody reaction was conducted overnight at 4 °C in an antibody solution containing a 100-fold dilution of anti-digoxigenin-POD and Fab fragments (Sigma–Aldrich) or a 500-fold dilution of anti-fluorescein-POD and Fab fragments (PerkinElmer) in blocking solution. After the antibody reaction, the sections were washed with TNT, and the TSA reaction was conducted using the TSA plus Biotin kit (Kiko Tech) for 30 min. After the TSA reaction, the sections were treated with streptavidin and Alexa Fluor™ 488 conjugate (Thermo Fisher) diluted 200-fold in blocking solution for 30 min and then sealed using VECTASHIELD mounting medium with DAPI (VECTOR). Sealed sections were observed for gene expression signals with an Axioplan fluorescence microscope (Carl Zeiss). All fluorescence photographs were taken using an Axiocam 503 color (Carl Zeiss) and adjusted for brightness and contrast in Adobe Photoshop.

In two-color FISH, hybridization was conducted in 2.5 ng/μl DIG and FITC-labeled RNA probe/hybridization solution overnight at 60 °C. After hybridization, the process from washing to antibody reaction was conducted as described above. After the antibody reaction, the sections were washed with TNT, and the TSA reaction was conducted using the TSA plus DIG kit (Kiko Tech) for 30 min. After the TSA reaction, the sections were treated with 15% H_2_O_2_/TBS for 30 min to inactivate the labeled digoxigenin of hybridized RNA probes. After blocking endogenous biotin, the antibody reaction was performed overnight at 4 °C in an antibody solution containing a 500-fold dilution of anti-fluorescein-POD and Fab fragments (PerkinElmer) and a 500-fold dilution of DyLight® 594 anti-digoxigenin (VECTOR) in blocking solution. The rest of the work was carried out in the same manner as described in single color FISH.

## Result

### The structure of the olfactory organ of bichir

The olfactory organs of the bichir were located in pairs on the left and right sides, each covered by cartilage and connected to the olfactory bulb (Fig. 1A, B). Environmental chemicals are detected by the olfactory organs as they flow from the anterior nostril tube toward the posterior nostril. The olfactory organ was subdivided into two organs, the main olfactory organ (MOO) and accessory olfactory organ (AOO). The AOO was located on the rostral side, and the MOO was located on the caudal side (Fig. 1 B; [34]). The MOO consisted of five tufts of lamellae, and the AOO consisted of two tufts. The lamellae of the MOO and AOO extended radially within tufts (Fig. 1C). The thick and thin epithelia lining the lamellae are sensory and nonsensory epithelium, respectively (Fig. 1D-G). The lamellae of the MOO and AOO are not connected to each other (Fig. 1C). Thus, it is obvious that the MOO and AOO are anatomically separated, reminding us of the idea that these two organs possess distinct functions; for example, they correspond to the MOE and VNO, as in tetrapods.

### DEG analyses between MOO and AOO of bichir

**Fig. 2:**
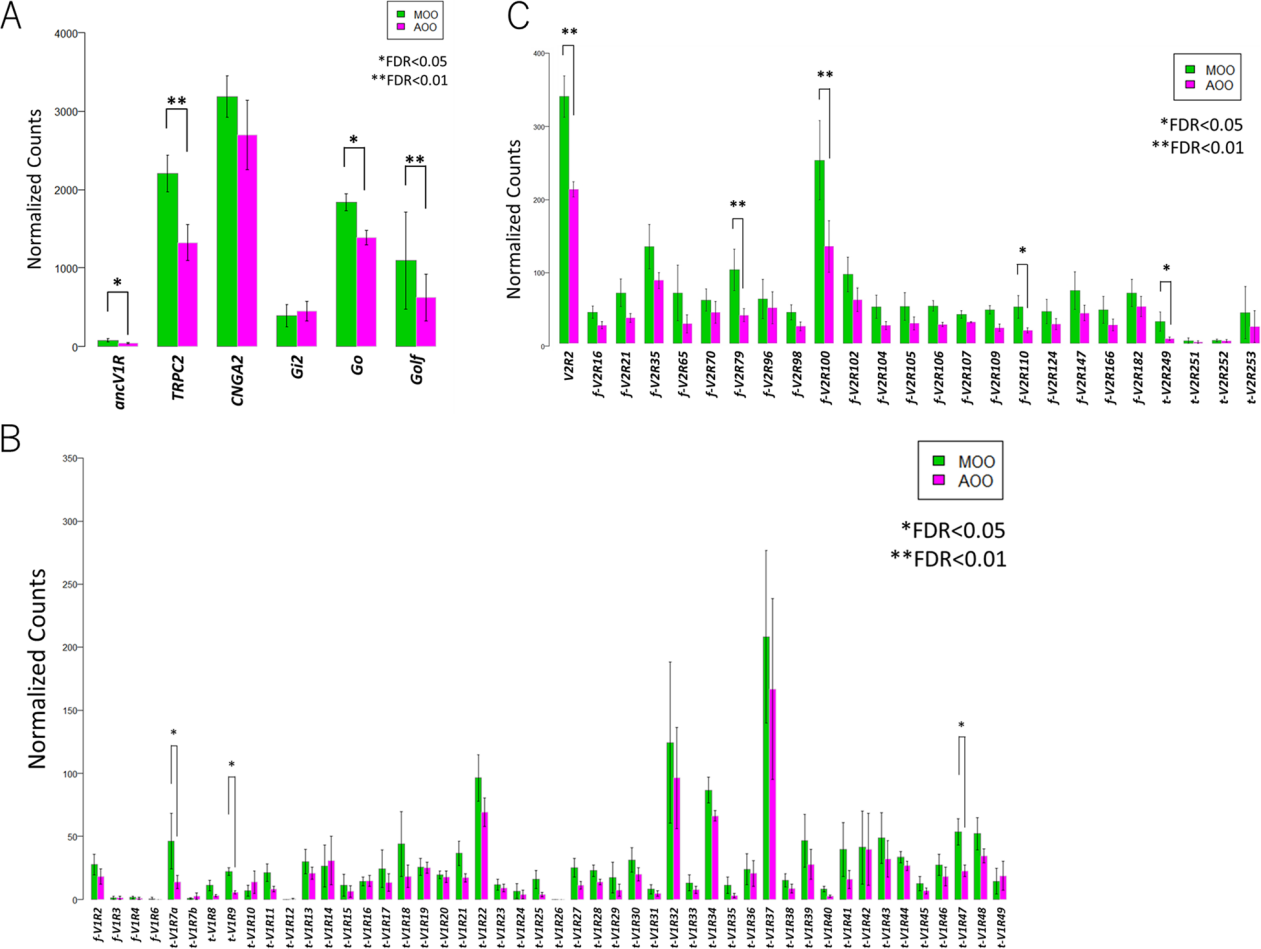
RNA-seq analysis of the MOO and AOO of bichir. Comparison of the expression levels of (**A**) several landmark genes related to the VNO and MOE, (**B**) *V1R*s and (**C**) *V2R*s. The vertical axis shows the normalized number of RNA-seq reads mapped to each gene region (RPM), and the horizontal axis shows gene names. Bars in green and magenta indicate the MOO and AOO, respectively (*: FDR < 0.05, **: FDR < 0.01)

To examine functional differences between the MOO and AOO, we conducted transcriptome analyses by comparing the number of each RNA sequence read of the MOO and AOO mapped to the reference genome data. Before the comprehensive DEG analysis, we first characterized several landmark genes. Then, vomeronasal-specific (*ancV1R*, *TRPC2*, *Gi2*, *Go*, *V1R*s, and *V2R*s) and olfactory-specific (*CNGA2* and *Golf*) genes were obtained to create an annotation file and compare the gene expression levels between MOO and AOO. Supplementary Fig. S1 shows the phylogenetic tree of the *V1R*s and *V2R*s of seven vertebrates. Both of these V1R and V2R trees confirmed that they were divided into two distinct clade groups, namely, “tetrapod-type” and “fish-type” ([25, 28] Zhang et al. 2021 in press). We first compared the gene expression levels of the landmark genes based on their normalized read counts. As a result, no clear differences, such as the presence or absence of the expression of particular genes, were observed, although the expression levels tended to be higher in the MOO than AOO (Fig. 2A). The expression of each V1R and V2R also did not show marked differences between the MOO and AOO groups (Fig. 2B, C). The above results suggest that MOO and AOO are not differentiated in terms of olfactory and vomeronasal function, as shown in tetrapods.

**Fig. 3:**
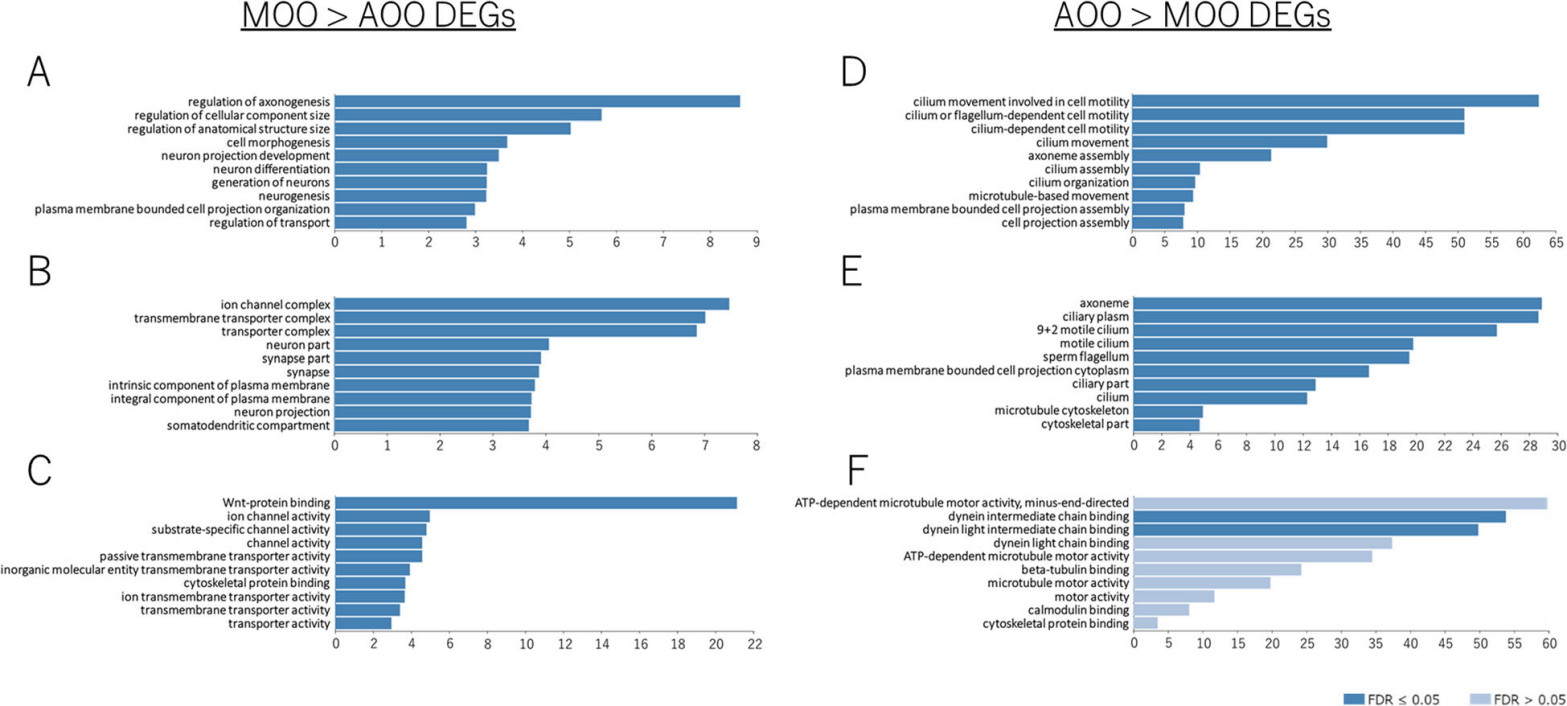
Gene ontology (GO) analyses of significant DEGs between the MOO and AOO of bichir. (**A**-**C**) and (**D**-**F**) indicate the GO terms of DEGs highly expressed in MOO and AOO, respectively. These results were obtained using WebGestalt. The vertical axis shows the gene categories, and the horizontal axis shows the enrichment rate (dark blue bar: FDR ≦ 0.05, light blue bar: FDR > 0.05)

Next, we conducted comprehensive DEG analyses and obtained 825 genes (FDR < 0.05), of which their expression levels were significantly different between MOO and AOO (MOO > AOO: 521, AOO > MOO: 304). The following GO analyses on these 825 genes using WebGestalt showed that DEGs of MOO > AOO were enriched in genes for the maintenance of neuronal (mainly axonal) morphology and neurotransmission, which may be caused by higher amounts of nerve bundles included in the MOO than in AOO (Fig. 3A-C). On the other hand, DEGs of AOO > MOO were enriched in genes for motile cilia (Fig. 3D-F). These results of the DEG analyses and comparison of the landmark genes suggest that both MOO and AOO are responsible for olfactory and vomeronasal functions, and AOO possesses an additional function in making water flow into the nasal cavity, which is important to accomplish efficient chemodetection in bichirs.

### Expression of vomeronasal genes in MOO and AOO of bichir

**Fig. 4:**
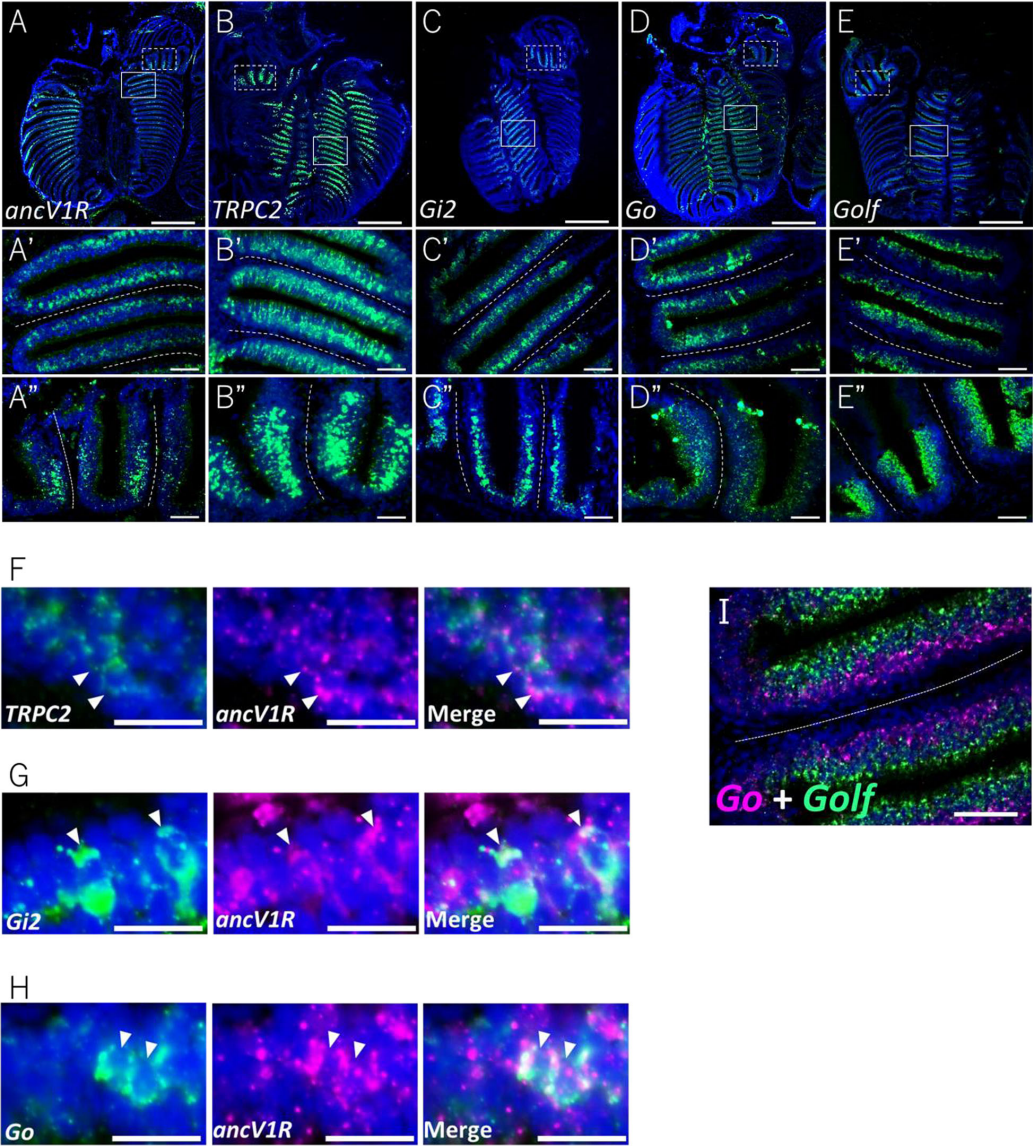
Overall views of gene expression patterns in the olfactory organ of bichir. (**A**-**E**) The expression patterns of four VNO-related genes (*ancV1R*, *TRPC2*, *Gi*_*2*_, *Go*) and MOE-related genes (*Golf*) in the olfactory lamellae. (A’-E’) Higher magnification views of the MOO in the solid squares in (**A**-**E**). (A”-E”) Higher magnification views of the lamellae of the AOO in the dotted squares in (**A**-**E**). Note that *ancV1R*, *TRPC2*, *Gi*_*2*_, and *Go* are expressed in the basal layer, while *Golf* is expressed in the apical layer of the lamellae. (**F**-**H**) Coexpression of *ancV1R* (magenta) with (**F**) *TRPC2*, (**G**) *Gi2*, and (**H**) *Go* (green). Arrowheads indicate the coexpressing cells. (**I**) Contrasting expression patterns of *Go* (magenta) in the basal layer and *Golf* (green) in the apical layer of the MOO lamellae confirmed by two-color FISH. The cell nuclei were stained by DAPI (blue). The dotted line indicates the center of the lamellae. The scale bars indicate 500 μm (**A**-**E**), 50 μm (A’-E’, A”-E”, I), or 20 μm (**F**-**H**)

Although the transcriptome analyses suggest that neither MOO nor AOO correspond to the VNO, as observed in tetrapods, it remains possible that these organs contain VNO-like regions with concentrated localizations of vomeronasal neurons. To examine the above possibility, we investigated the expression patterns of the landmark genes of the VNO in the olfactory organ of bichir at the cellular level by FISH. As shown in Fig. 4, *ancV1R*, *TRPC2*, *Gi2*, and *Go* were expressed in the basal layers of the olfactory lamellae of the MOO and AOO (Fig. 4A-D, A’-D′, A”-D″). On the other hand, *Golf*, the landmark gene for MOE, was expressed in the apical layer of the lamellae of the MOO and AOO (Fig. 4E, E‘, E”, I). It is worth noting that the patterns of expression of the landmark genes of the VNO were unlocalized but rather broad across the basal layer of the lamellae.

**Fig. 5:**
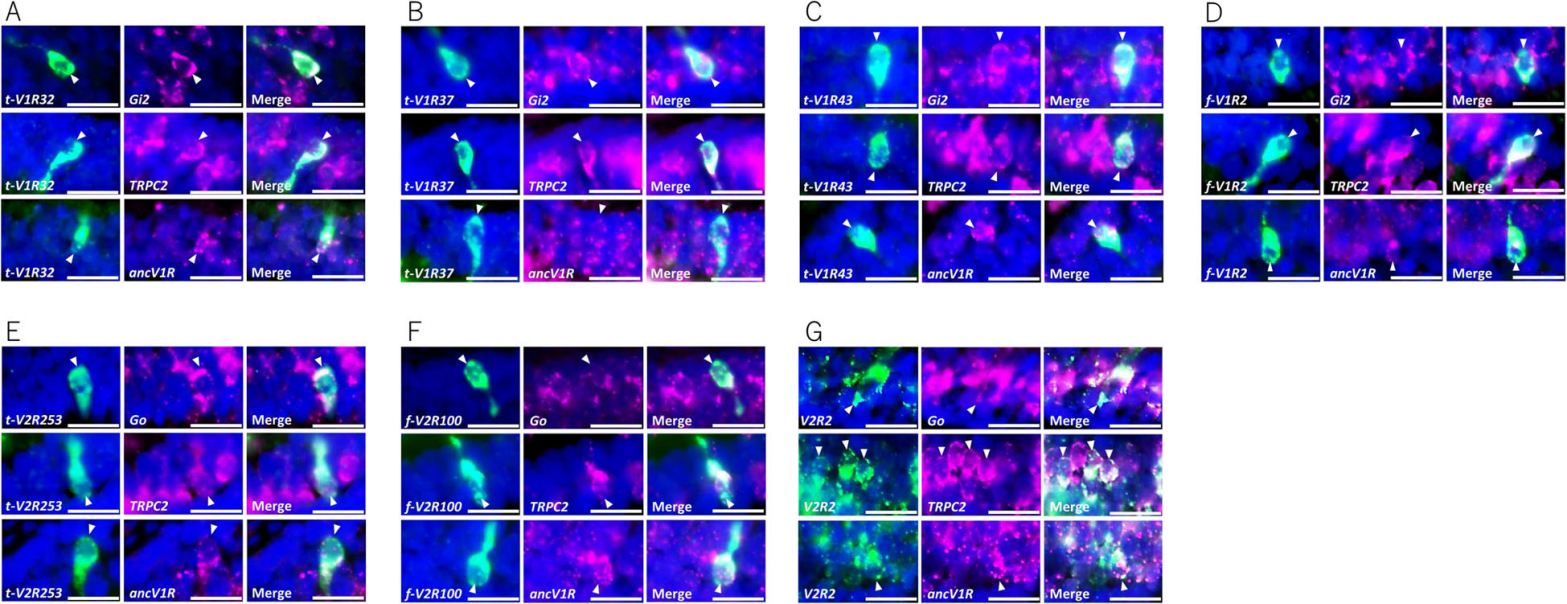
Coexpression of *V1R*s and *V2R*s with VNO-related genes in the olfactory sensory neurons of bichir. (**A**-**D**) Coexpression of (**A**) t-*V1R32*, (**B**) t-*V1R37*, (**C**) t-*V1R43*, and (**D**) f-*V1R2* (green) with VNO-related genes (*Gi*_*2*_, *TRPC2*, and *ancV1R*) (magenta). (**E**-**G**) Coexpression of (**E**) t-*V2R*, (F) f-*V2R*, and (**G**) *V2R2* (green) with VNO-related genes (*Go*, *TRPC2*, and *ancV1R*) (magenta). Arrowheads indicate the coexpressing cells. The cell nuclei were stained by DAPI (blue). All scale bars indicate 20 μm

Since the landmark genes of the VNO were all expressed in the MOO and AOO, we next examined whether the coexpression patterns in bichir were similar to those of tetrapods. Two color FISH using the combinations of the probes of *ancV1R*, *TRPC2*, *Gi2*, and *Go* showed coexpression patterns in all cases: *ancV1R*-*TRPC2*, *ancV1R*-*Gi2*, and *ancV1R*-*Go* (Fig. 4F-H). To investigate the characteristics of the vomeronasal sensory neurons in bichir in more detail, we examined the coexpression of each V1R, *t-*V1R (*t-V1R 32*, 34, *37*, *43*, and *49*) and *f-V1R* (*f-V1R2*, *3*, *4*, and *6*) (Supplementary Fig. S1 A, Fig. 2B), with *ancV1R*, *TRPC2*, and *Gi2*. The results of two-color FISH showed that *t-V1R32*,*37*,*43*, and *f-V1R2* were coexpressed with *ancV1R*, *TRPC2*, and *Gi2* (Fig. 5A-D, Supplementary Fig. S2A-C). Signals were not detected for *t-V1R34*, *49*, *f-V1R3*, *4*, and *6* (data not shown), which may be due to the low levels of their expression. We also examined the coexpression of each V2R, *t-V2R253*, *f-V2R100*, and *V2R2* (Supplementary Fig. S1B, Fig. 2C), with *ancV1R*, *TRPC2*, and *Go*. The examined genes showed coexpression in all cases (Fig. 5E-G). These lines of coexpression data revealed that typical vomeronasal sensory systems of *V1R*s and *V2R*s, including their signaling cascades, which were originally characterized in tetrapods, all function in the MOO and AOO of bichir.

It is implicative to note that the ratios of coexpression with *Gi*_*2*_ vary between *t-V1R* and *f-V1R*. Specifically, almost all t-*V1R37*-expressing cells coexpressed *Gi*_*2*_ (98.8%), whereas less than half of *f-V1R2*-expressing cells coexpressed *Gi*_*2*_ (41.7%) (Table 1). *TRPC2* was equally coexpressed with *f-V1R* (95.3%) and *t-V1R* (96.5%). The above results imply that some unidentified G proteins couple with *f-V1R*s in the olfactory organ of bichir.

**Table 1:** Coexpression ratios of *V1R* and *V2R* genes and VNO-related genes in bichir olfactory organs

	TRPC2	Gi2	Go
f-V1R2	95.3% (82/86)^a^	41.7% (30/72)	-
t-V1R37	96.5% (276/286)	98.8% (480/486)	-
f-V2R100	88.6% (156/176)	-	87.0% (208/239)
t-V2R253	86.2% (112/130)	-	84.4% (233/276)

^a^The actual numbers of the co-expression cells observed in the 3-7 sections were indicated in parentheses

### Expression of *ancV1R* in lungfish and spotted gar

**Fig. 6:**
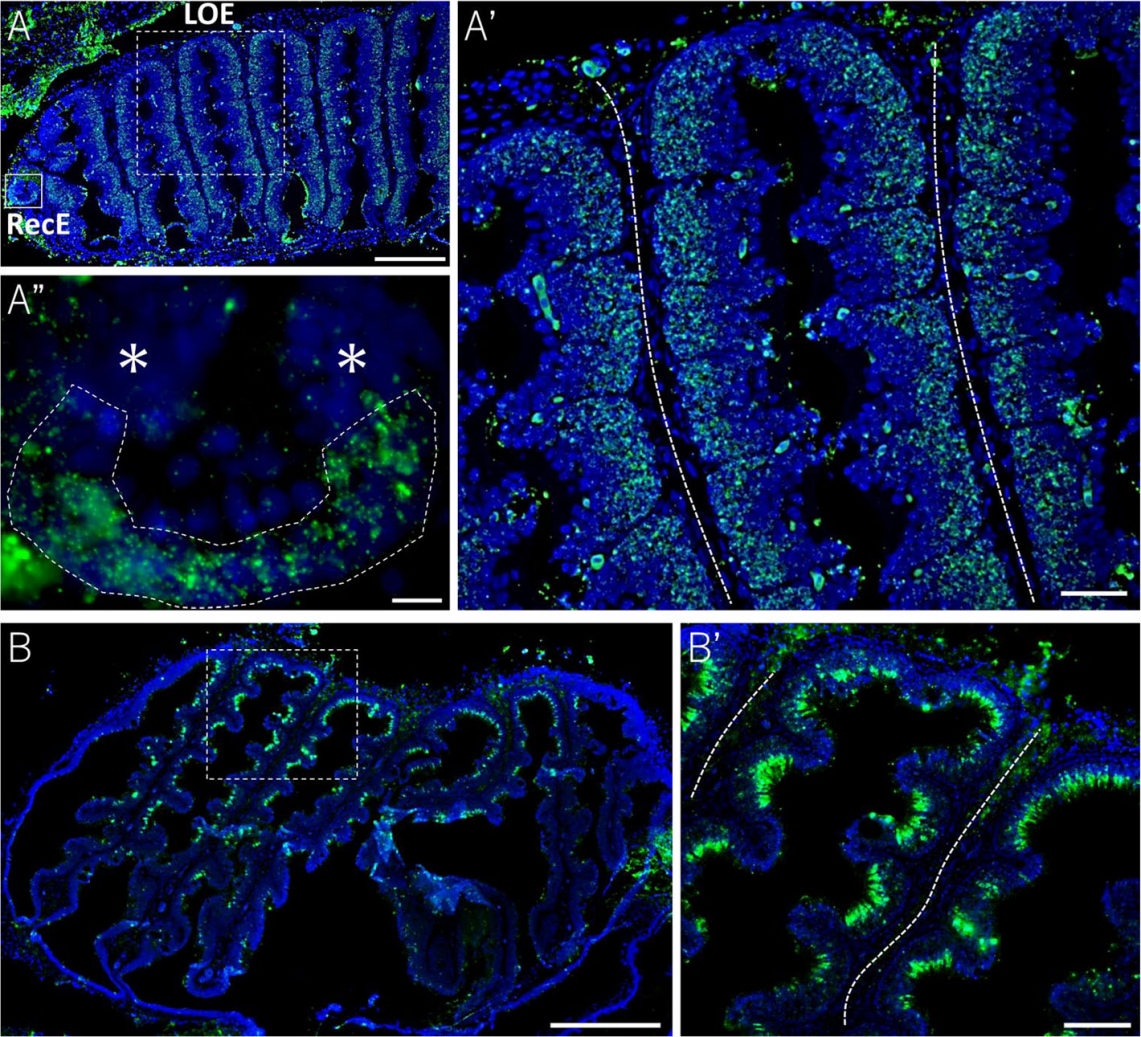
Expression pattern of *ancV1R* in lungfish and gar. (**A**) The expression pattern of *ancV1R* (green) in a sagittal section of the olfactory organ of lungfish. The dotted and solid squares indicate LOE and RecE, respectively. (A’) High magnification view of the dotted square in (A). Note that *ancV1R* is expressed in the basal layer of the LOE. (A”) Higher magnification view of the solid square in (A). *ancV1R* is expressed in the sensory epithelium of the RecE surrounded by a dotted line. Asterisks indicate the location of the nonsensory epithelium. (**B**) The expression pattern of *OMP* in a horizontal section of the olfactory organ of spotted gar. (B′) Higher magnification of the dotted square in (B). Note that *ancV1R* is expressed by most sensory neurons scattered in the concave regions of the lamella. The dotted line indicates the center of the lamellae. The scale bars indicate 500 μm (A, B), 100 μm (A’, B′), or 20 μm (A”)

**Fig. 7:**
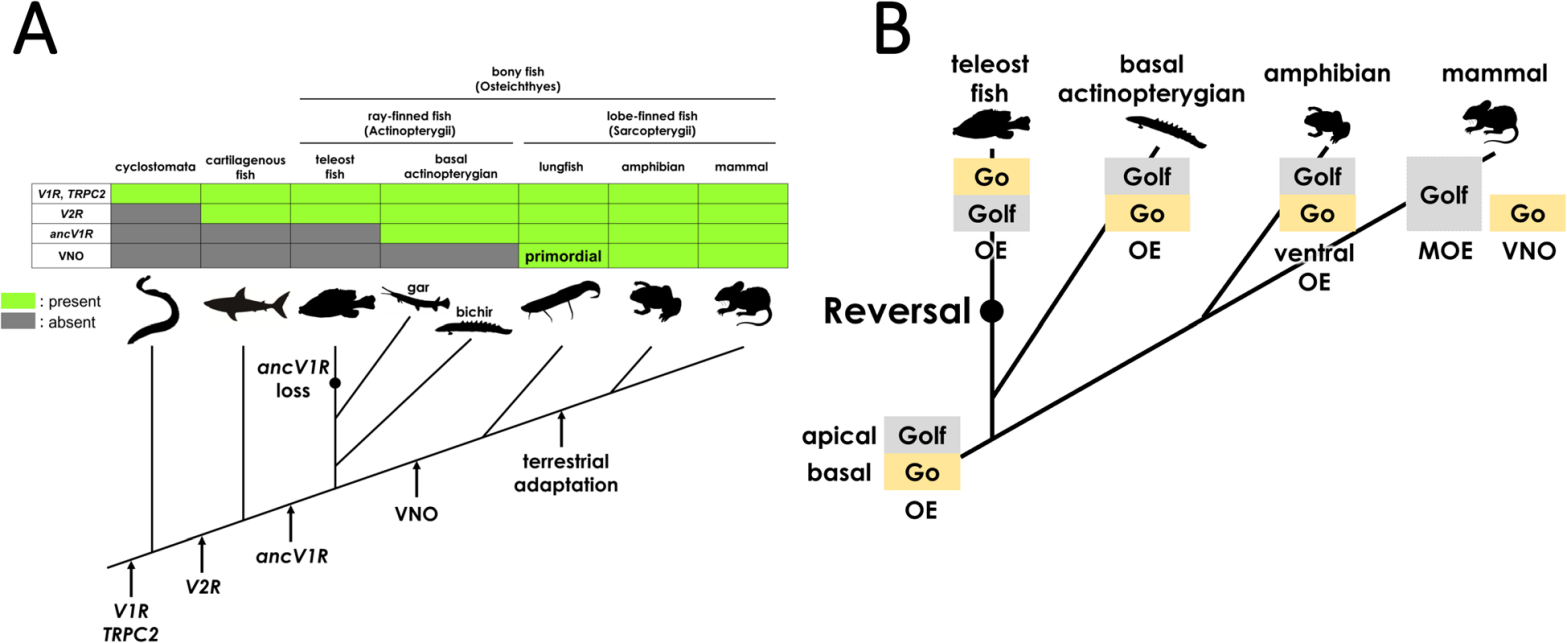
The origin and evolution of the VNO in vertebrates. (**A**) Possible scenarios for the timing of VNO acquisition and a step-by-step evolution of the vomeronasal system in vertebrates. Considering that the VNO-like region was not found in bichir and gar, the acquisition of the VNO is likely to be later than the common ancestor of Sarcopterygii. However, the gene sets for advanced vomeronasal sensory neurons had already existed in the common ancestor of Osteichthyes. (**B**) Reversal in the pattern of *Go*/*Golf* expressions. Considering that the pattern of expression of *Go* (basal) and *Golf* (apical) in the olfactory epithelium of bichir is similar to that of amphibians (and partly similar to the VNO of mammals), it is parsimonious that the reversal of *Go*/*Golf* expression occurred in the common ancestor of teleost fish

In addition to bichir, we investigated the presence/absence of the primordial VNO in so-called ancient fish, such as lungfish and spotted gar, by using the newly identified marker for the vomeronasal sensory neuron *ancV1R* [29, 43]. First, we examined the expression pattern of *ancV1R* in the olfactory organ of lungfish. FISH of the olfactory organ of lungfish showed that *ancV1R* was expressed in RecE as well as in the basal layer of the lamella olfactory epithelium (LOE) (Fig. 6A, A’, A”). These results support the hypothesis that RecE is a primordial VNO [6–8]. The expression pattern of *ancV1R* in the basal layer of LOE of lungfish was similar to that observed in the MOO and AOO of bichir. We additionally examined the pattern of *ancV1R* expression in the olfactory organ of spotted gar, which is a basal group of Actinopterygii but diverged later than bichir during evolution (Fig. 7A). FISH of the olfactory organ of spotted gar showed that *ancV1R* was expressed in the concave regions of the olfactory lamella (Fig. 6B, B’). To characterize the distribution of cell types in the olfactory organ of the spotted gar, we next examined the expression pattern of *OMP*, which is expressed throughout olfactory sensory neurons [13]. The *OMP* was also shown to be expressed in the same concave region as that observed in *ancV1R* (Supplementary Fig. S3A, A’). Thus, the expression pattern of *ancV1R* suggests that the olfactory organ of the spotted gar is undifferentiated in that vomeronasal sensory neurons are not concentrated in a particular region but are scattered throughout the sensory epithelium of the olfactory lamellae.

## Discussion

### Re-evaluation of AOO of bichir

Transcriptomic studies, including comparisons of several landmark genes, DEGs and FISH analyses, suggest that MOO and AOO, which are anatomically separated, were not functionally differentiated in terms of pheromone and odorant detection. However, DEG analyses suggest that AOO may have a different function in addition to chemoreception. Specifically, the DEGs in the AOO were enriched for genes related to motile cilia, which may be used for making water flow into the nasal cavity. The olfactory organ of bichir is connected to the external environment through a long nasal tube and consists of several tufts of lamellae, which fundamentally differ from that of the teleost and resemble that of coelacanth [34]. Because bichir has such a distinctive morphology in its olfactory organ, a strong ventilation system would be necessary to create sufficient water flow to the olfactory epithelium. Indeed, motile cilia were observed in the nasal pit of zebrafish [44]. These motile cilia were shown to be used for generating flow fields in the olfactory epithelium, which increase the sensitivity and temporal resolution of chemical stimuli. It is likely that bichirs, which largely depend on the chemical cues for reproduction and feeding [34], use a strong ventilation system enabled by AOO. To fully understand the possible role of AOO in bichir, more detailed investigation based on hydrodynamics and behavioral analyses will be indispensable in the near future. Given that the developmental process of AOO and MOO from olfactory placodes in bichir is still poorly understood, it is a matter of further debate whether the developmental process of AOO shares commonality with the VNO and RecE.

### The origin and evolution of vomeronasal system

In this study, RNA-seq and FISH analyses revealed that no VNO-like region, the concentration of vomeronasal neurons, was found in the olfactory organ of bichir. This result shows that the olfactory organ of bichir is more similar to that of teleost fish rather than to that of lobe-finned fish (lungfish and tetrapods) in the context of the presence/absence of the VNO, reflecting its ancestral position in the phylogenetic tree of bony vertebrates (Fig. 7A). However, our detailed FISH analyses and the repertoire of vomeronasal gene components in bichir simultaneously provide important insight into understanding a step-by-step evolution of vomeronasal sensory neurons from jawless vertebrates to tetrapods, leading to the emergence of the VNO.

In lampreys, the genetic components of vomeronasal neurons (*f-V1R*, *TRPC2*) were already present [24]. However, the subsequent acquisition of additional components in the common ancestor of jawed vertebrates (*V2R*s) and that of bony vertebrates (*ancV1R*, t-*V1R*, t-*V2R*) may have led to the acquisition of more advanced vomeronasal neurons observed in tetrapods ([25] Zhang et al. 2021 in press). In bichir, we showed that *ancV1R-TRPC2-V1Rs-Gi*_*2*_ coexpressing and *ancV1R-TRPC2-V2Rs-Go* coexpressing neurons were both distributed in the basal layer of the MOO and AOO, which exactly corresponded to the neurons in the basal layer of the LOE of lungfish. In contrast, teleost fish lost several genetic components of the vomeronasal system, *ancV1R*, *t-V1Rs*, and *t-V2Rs*. Taking the repertoire of the genetic components into consideration, vomeronasal neurons of bichir are more similar to those of lungfish and tetrapods than those of lamprey, shark and teleost fish. It is worth noting that although bichir possesses both *t-V1Rs* and *f-V1Rs*, the ratio of coexpression with *Gi*_*2*_ is higher in *t-*V1R-expressing neurons than in *f-V1Rs* (Table 1), implying that *t-V1R-Gi*_*2*_ coexpressing neurons are more advanced vomeronasal neurons observed in tetrapods. Indeed, in teleost fishes, *f-V1Rs* were not coexpressed with *Gi*_*2*_ in the olfactory organ [45]. In lungfish, *ancV1R-TRPC2-V2Rs-Go* coexpressing vomeronasal neurons concentrate to form the RecE ([6–8], this study), which is likely to be the origin of a primordial VNO. This localized coexpression of *ancV1R-TRPC2-V2Rs-Go* was retained in the amphibian VNO after terrestrial adaptation [22, 29, 46, 47].

In addition to the expression of landmark genes in vomeronasal neurons, bichir is more similar to lungfish and tetrapods than to teleost fish in terms of the arrangement of layers for *Go-* and *Golf*-expressing neurons in the olfactory lamellae. In teleosts, *V2R*-*Go*-expressing and *OR*-*Golf*-expressing neurons were observed in the apical and basal layers of the OE, respectively [13, 45, 48, 49]. However, in amphibians (newt and frogs), *V2R*-*Go*-expressing and *OR*-*Golf*-expressing neurons were observed in the basal and apical layers of the ventral OE, respectively. In mammals (mice), *OR*-*Golf*-expressing neurons were observed in the entire layer of the MOE, and *V2R*-*Go*-expressing and *V1R*-*Gi2*-expressing neurons were observed in the basal and apical layers of the VNO, respectively. Namely, the distributions of the *V2R*-*Go-* and *OR*-*Golf*-expressing layers of amphibians and the *V2R*-*Go*-expressing layer of mammals are opposite to those of zebrafish [22, 23, 50]. Therefore, it was proposed that the reversal of the layers for *V2R*-*Go*- and *OR*-*Golf*-expressing neurons occurred in the timing of terrestrial adaptation of vertebrates. However, in bichir, we found that *Golf*-expressing and *Go*-expressing neurons were each distributed in apical and basal layers, respectively, suggesting that the reversal of the layer had occurred in the common ancestor of teleost fishes (Fig. 7B).

## Conclusion

In this study, our transcriptome analyses provided crucial insights into the evolution of the olfactory organ of bichir at two major points. First, the MOO and AOO of bichir are not functionally differentiated in terms of chemical detection but are differentiated in that AOO may possess additional function in making water flow into the nasal cavity. Second, although the VNO was not found in the olfactory organ of bichir, the expression pattern of landmark genes suggested that the vomeronasal neurons of bichir are more similar to those of lungfish and tetrapods than to those of sharks and teleost fishes. The findings suggested that advanced tetrapod-like vomeronasal sensory neurons have already been present, at least in the common ancestor of bony vertebrates. Because previous studies have been limited to teleost fishes and tetrapods, the evolutionary process of vomeronasal neurons has not been reliably described. However, the genomes of bony fishes and basal ray-finned fishes, which became successively available, may further illuminate the detailed evolutionary history of vomero-olfactory systems in vertebrates from fish to mammals.

## Supplementary Information

**Additional file 1 **The online version contains supplementary materials. **Supplementary Fig. S1.** Phylogenetic tree of *V1R*s and *V2R*s of seven vertebrate species. The OTU names of the seven vertebrates are as follows: Pse, bichir; Loc, spotted ger; Ola, medaka; Hch or Oni, cichlid; Dre, zebrafish; Lch, coelacanth; Xtr, tropical clawed frog; and Bta, cow. Bootstrap values are only partially shown above each branch. Scale bars indicate the number of amino acid substitutions per site. (A) The phylogenetic tree of *V1R*s. *T2R*s (bitter-taste receptors) were used as the outgroup. Bichir *V1R*s are shown in magenta, indicating the presence of t-*V1R*s, f-*V1R*s, and *ancV1R*. (B) The phylogenetic tree of *V2*s. *CaSR*s (calcium sensing receptors) were used as the outgroup. Bichir *V2R*s are shown in magenta, indicating the presence of t-*V2R*s, f-*V2R*s, *V2R2*, and *ancV2R*. **Supplementary Fig. S2.** Coexpression of t-*V1R*s with *ancV1R* in the olfactory organ of bichir. (A) t-*V1R11*, (B) t-*V1R22*, and (C) t-*V1R30* (green) are coexpressed with *ancV1R* (magenta). The cell nuclei were stained by DAPI (blue). Arrowheads indicate the coexpressing cells. All scale bars represent 20 μm. **Supplementary Fig. S3.** *OMP* expression pattern in the olfactory organ of spotted gar. (A) The expression pattern of *OMP* (green) in a horizontal section of the spotted gar olfactory organ. (A’) Higher magnification view of the dotted square in (A). Note that *OMP* is expressed only in the concave areas of the lamellae, suggesting that these concave areas are sensory epithelium and that the convex areas are nonsensory epithelium. The dotted lines indicate the center of the lamellae. The cell nuclei were stained by DAPI (blue). The scale bars indicate 500 μm (A) or 100 μm (A’). **Supplementary Table. S1. **PCR primers used in this study.

## Data Availability

All sequence reads were deposited in the DDBJ Sequence Read Archive under accession no. PRJDB12173.

## Abbreviations

OE: Olfactory epithelium
MOE: Main olfactory epithelium
VNO: Vomeronasal organ
MOO: Main olfactory organ
AOO: Accessory olfactory organ
DEG: Differentially expressed gene
V1R: Vomeronasal receptor type I
V2R: Vomeronasal receptor type II
OR: olfactory receptor
TRPC2: Transient receptor potential cation channel subfamily C member 2
OMP: Olfactory marker protein
