# Supplementary material for: A transcriptomic reevaluation of the accessory olfactory organ in Bichir (Polypterus senegalus)
Source: Zoological Lett. 2022 Feb 8;8:5. doi: 10.1186/s40851-022-00189-z (PMC8822828; doi:10.1186/s40851-022-00189-z)
Supplement: Supplementary file 1 [file 40851_2022_189_MOESM1_ESM.docx]

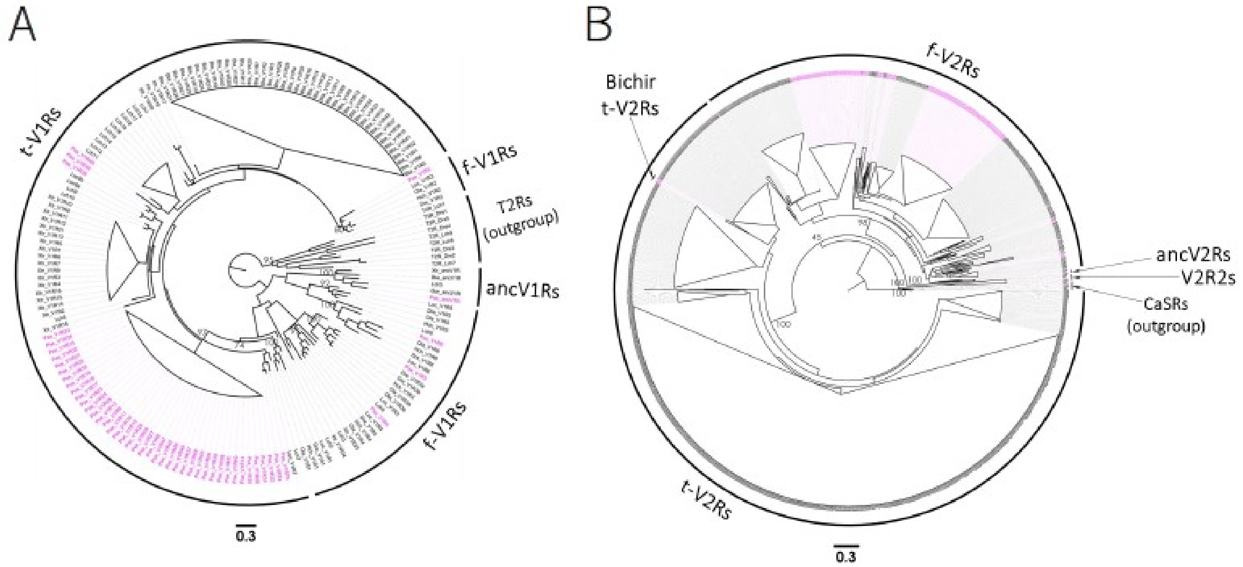

Supplementary Fig. S1

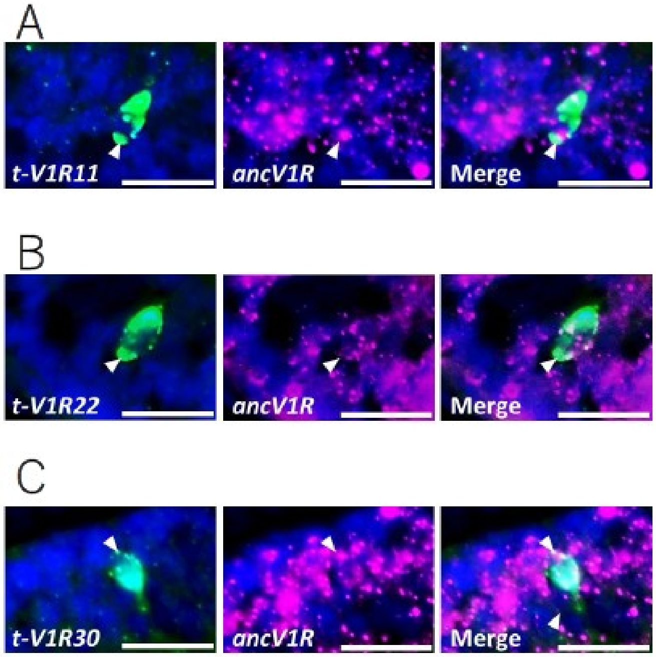

Supplementary Fig. S2

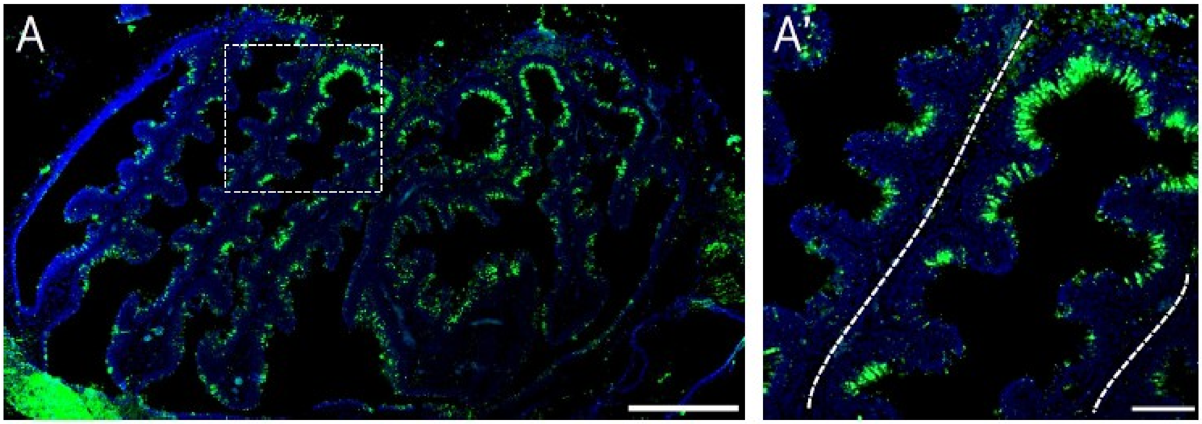

Supplementary Fig. S3
